# Polymorphisms of the cryptochrome 2 and mitoguardin 2 genes are associated with the variation of lipid-related traits in Duroc pigs

**DOI:** 10.1038/s41598-019-45108-z

**Published:** 2019-06-21

**Authors:** Emilio Mármol-Sánchez, Raquel Quintanilla, Taina F. Cardoso, Jordi Jordana Vidal, Marcel Amills

**Affiliations:** 1 Department of Animal Genetics, Centre for Research in Agricultural Genomics (CRAG), CSIC-IRTA-UAB-UB, Campus de la Universitat Autònoma de Barcelona, Bellaterra, Spain; 2 Animal Breeding and Genetics Programme, Institute for Research and Technology in Food and Agriculture (IRTA), Torre Marimon, Caldes de Montbui, Spain; 3 CAPES Foundation, Ministry of Education of Brazil, Brasilia, D. F. Brazil; 4 Departament de Ciència Animal i dels Aliments, Universitat Autònoma de Barcelona, Bellaterra, Spain

**Keywords:** Gene expression, Genetic markers

## Abstract

The genetic factors determining the phenotypic variation of porcine fatness phenotypes are still largely unknown. We investigated whether the polymorphism of eight genes (*MIGA2*, *CRY2*, *NPAS2*, *CIART*, *ARNTL2*, *PER1*, *PER2* and *PCK1*), which display differential expression in the skeletal muscle of fasted and fed sows, is associated with the variation of lipid and mRNA expression phenotypes in Duroc pigs. The performance of an association analysis with the GEMMA software demonstrated that the rs330779504 SNP in the *MIGA2* gene is associated with LDL concentration at 190 days (LDL_2_, corrected *P*-value = 0.057). Moreover, the rs320439526 SNP of the *CRY2* gene displayed a significant association with stearic acid content in the *longissimus dorsi* muscle (LD C18:0, corrected *P*-value = 0.015). Both SNPs were also associated with the mRNA levels of the corresponding genes in the *gluteus medius* skeletal muscle. From a biological perspective these results are meaningful because *MIGA2* protein plays an essential role in mitochondrial fusion, a process tightly connected with the energy status of the cell, while *CRY2* is a fundamental component of the circadian clock. However, inclusion of these two SNPs in chromosome-wide association analyses demonstrated that they are not located at the peaks of significance for the two traits under study (LDL_2_ for rs330779504 and LD C18:0 for rs320439526), thus implying that these two SNPs do not have causal effects.

## Introduction

The genome-wide analysis of gene expression data obtained from RNA-seq experiments can provide valuable information in order to understand the biology of production phenotypes and how they are genetically regulated. Cardoso *et al*.^1^ compared the muscle transcriptomic profiles of Duroc sows before and after feeding and, in doing so, they demonstrated that the ingestion of food is associated with changes in the mRNA levels of several circadian genes including the cryptochrome circadian regulator 2 (*CRY2*), neuronal PAS domain protein 2 (*NPAS2*), circadian associated repressor of transcription (*CIART*), aryl hydrocarbon receptor nuclear translocator like 2 (*ARNTL2*), period circadian regulator 1 (*PER1*) and period circadian regulator 2 (*PER2*). The identification of circadian clock regulator genes is particularly relevant because they have been broadly reported as major contributors to lipid metabolism and energy homeostasis^2–7^, driving changes in the expression of multiple transcripts and modulating cell response to different stimuli such as food intake^5,8,9^. Two other interesting genes identified by Cardoso *et al*.^1^ as differentially expressed before and after eating were mitoguardin 2 (*MIGA2*), which regulates mitochondrial fusion^10^, a process tightly connected with energy homeostasis^11^, and phosphoenolpyruvate carboxykinase 1 (*PCK1*), an enzyme fundamental for the maintenance of glucose and lipid levels^12^.

The expression of the eight genes mentioned above (*ARNTL2*, *CIART*, *CRY2*, *NPAS2*, *PER1*, *PER2*, *PCK1* and *MIGA2*) is affected by food intake and there is ample evidence that they have a key role in carbohydrate and lipid metabolism^8,10,13–15^. The main hypothesis that we aim to test in the current work is whether the variability of these eight genes is associated with lipid phenotypes recorded in a Duroc pig population denominated as Lipgen (Supplementary Table 1). To achieve this goal, we have first identified a total of 20 polymorphisms (Table 1) in these eight genes by using a previously published RNA-Seq data set corresponding to 52 pigs from the Lipgen population^16^. These 20 SNPs have been genotyped in 345 pigs from the Lipgen population with available records for a broad array of lipid traits listed in Supplementary Table 1, *i*.*e*. serum lipid concentrations^17,18^, *longissimus dorsi* (LD) and *gluteus medius* (GM) muscle fatty acid composition^19^ and backfat thickness. Subsequently, those SNPs showing significant associations (after correction for multiple testing) with a given lipid trait, have been further studied by investigating if they are associated with gene expression as well as by performing chromosome-wide association analyses based on Porcine SNP60 BeadChip data. The liver and GM muscle mRNA expression data sets^18,20^ and the Porcine SNP60 BeadChip genotypes^18,21^ used for this purpose were generated in previous studies (Supplementary Table 2).

**Table 1:** List of polymorphisms genotyped in a population of Duroc pigs (N = 345). SSC: pig chromosome.

Gene	Sscrofa.11.1					
	SSC	Start	End	Strand	SNP	Effect
*MIGA2*	1	269338125	269362328	+	rs322533788	Splice region
					rs80923452	Splice region
					rs80832336	Splice region
					rs330779504	Splice region
*CRY2*	2	16584431	16620669	−	rs320439526	5′-UTR
*NPAS2*	3	53295744	53418480	−	rs335603631	Missense
*CIART*	4	98797378	98814553	−	rs322666984	Missense
*ARNTL2*	5	46330522	46482280	−	rs326158774	Splice region
*PER1*	12	53341329	53376723	−	rs699427837	Missense
					rs345340955	Splice region
					rs81436952	Missense
*PER2*	15	137793421	137836268	−	rs344440225	Splice region
					rs329662925	Missense
					rs324793161	Missense
					rs80910874	Missense
					rs325502974	Missense
*PCK1*	17	57930356	57938817	+	rs343196765	Missense
					rs331782052	Missense
					rs345064848	Splice region
					rs320568163	Missense

## Results

### Association analyses for lipid traits

**Figure 1:**
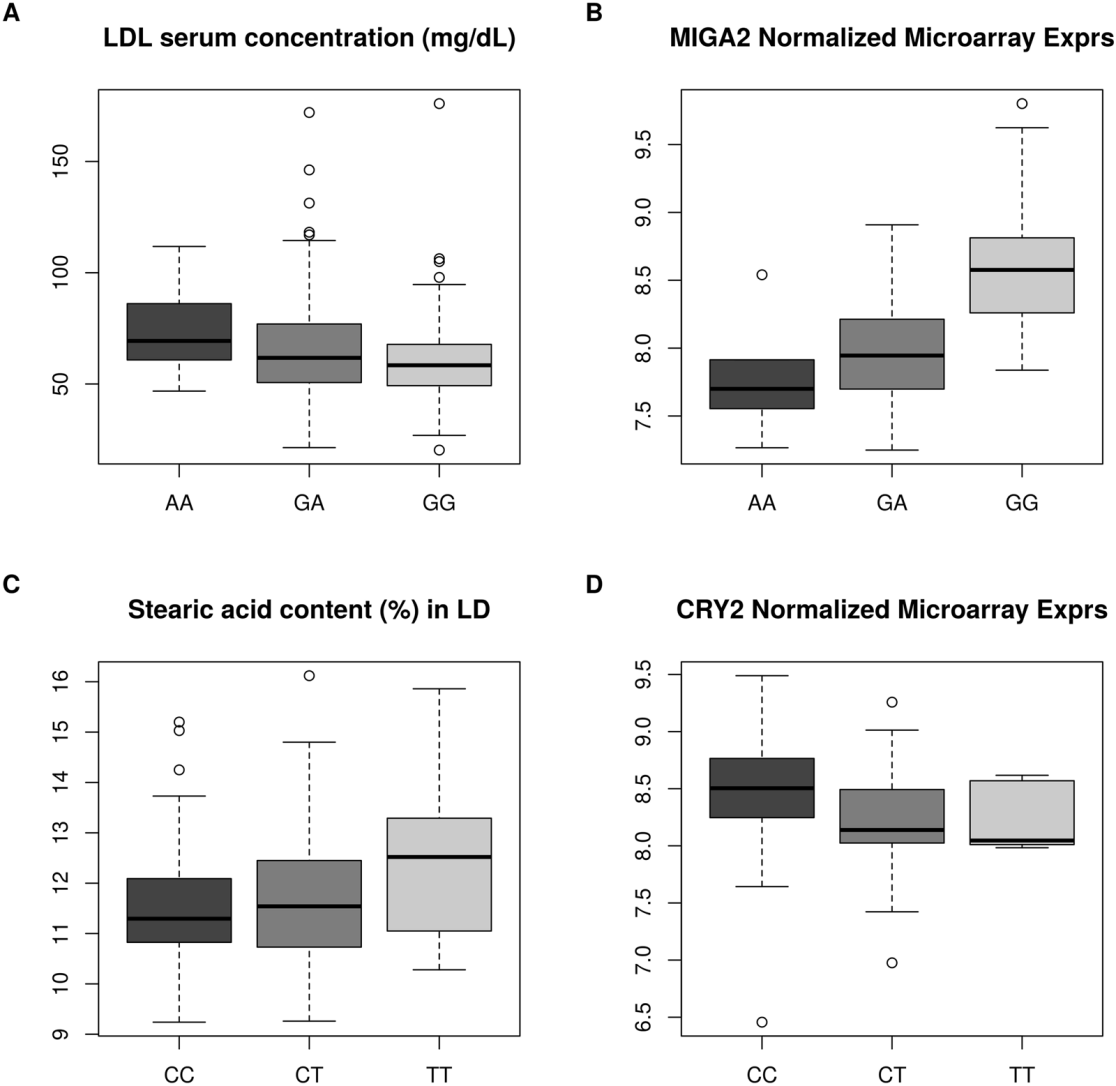
(**A**) Boxplots depicting the median and the distribution of serum low density lipoprotein concentrations at ~190 days for each one of the three rs330779504 genotypes: GG (N = 191), GA (N = 125) and AA (N = 16). (**B**) Boxplots depicting the median and the distribution of *MIGA2* mRNA expression levels in the *gluteus medius* skeletal muscle for each one of the three rs330779504 genotypes: GG (N = 48), GA (N = 33) and AA (N = 6). (**C**) Boxplots depicting the median and the distribution of stearic acid (C18:0) content in LD skeletal muscle for each one of the three rs320439526 genotypes: CC (N = 135), CT (N = 161) and TT (N = 37). **(D)** Boxplots depicting the median and the distribution of *CRY2* mRNA expression levels in the *gluteus medius* skeletal muscle for each one of the three rs320439526 genotypes: CC (N = 37), CT (N = 45) and TT (N = 6).

Previous data sets employed for making the association analyses with a wide variety of lipid-related traits are listed in Supplementary Table 2. Performance of association analyses between the 20 selected SNPs and the phenotypes listed in Supplementary Table 1 allowed us to identify several associations that were significant at the nominal level (Table 2). Three SNPs in the *PER1* gene were associated with LD and GM C18:3, and there was also an association between the *CIART* genotype and backfat thickness. Two SNPs in the *PCK1* gene were associated with LD C17:0, and *CRY2* and *MIGA2* genotypes showed associations with several serum lipid and fatty acid composition traits. These results were consistent with the relevant role of the genes under study on metabolism and energy homeostasis. However, only two associations remained significant after correction for multiple testing. The serum concentration of low-density-lipoproteins (LDL) measured at ~190 days was significantly associated with the rs330779504 SNP (Table 2), a splice region variant located in the beginning of intron 14 (1:269.360 Mb) of the mitoguardin 2 gene (*MIGA2*). Pigs inheriting the A-allele showed an increased LDL cholesterol concentration (Fig. 1A), with homozygous AA animals having a higher median blood LDL concentration (69.35 mg/dL) than GA (61.75 mg/dL) and GG (58.40 mg/dL) individuals. Kruskal-Wallis ranking test for differences in median LDL concentrations yielded a *P*-value of 5.14E-03 (Supplementary Table 3), thus supporting the existence of significant differences among the three rs330779504 genotypes. Besides, this *MIGA2* polymorphism also displayed an additive effect on palmitic acid content in LD muscle, total serum cholesterol concentration at ~190 days of age and the ratio between omega-6 and omega-3 desaturation in LD, but only at the nominal *P*-value level of significance (Table 2). The proportion of variance in LDL cholesterol concentration explained by rs330779504 genotype was 2.16% (SE = 0.03%).

**Table 2:** Polymorphisms significantly associated with lipid-related traits^a^.

Gene	SNP	Type	Trait	*P*-value	*q*-value	δ (SE)	A_1_	MAF
***MIGA2***	**rs330779504 (1:269.360 Mb)**	**Splice region variant (G/A)**	**LDL**_**2**_	**2**.**71E-03**	**5**.**69E-02**	**6**.**26** (**2**.**01)**	**A**	**0**.**236**
			LD (C16:0)	7.89E-03	1.66E-01	−0.43 (0.14)		
			TotalCholest_2_	3.06E-02	2.73E-01	5.86 (2.53)		
			LDFAn6/FAn3	2.91E-02	6.10E-01	−1.00 (0.42)		
	rs80832336 (1:269.359 Mb)	Splice region variant (C/T)	LD (C16:0)	7.06E-02	3.63E-01	−0.24 (0.13)	T	0.381
			TotalCholest_2_	2.69E-02	2.73E-01	5.03 (2.13)		
	rs322533788 (1:269.341 Mb)	Splice region variant (T/C)	GM (C10:0)	3.77E-02	4.32E-01	−0.01 (0.01)	C	0.093
			GM (C20:0)	5.46E-03	1.15E-01	−0.04 (0.01)		
***CRY2***	**rs320439526 (2:16.620 Mb)**	**5′-UTR variant (C/T)**	**LD (C18:0)**	**7**.**04E-04**	**1**.**48E-02**	**0**.**39** (**0**.**12)**	**T**	**0**.**353**
			TotalCholest_2_	4.22E-02	2.73E-01	−4.74 (2.23)		
			LDUFA	3.10E-02	3.82E-01	−0.43 (0.20)		
			LDSFA	3.69E-02	3.75E-01	0.42 (0.20)		
			LD (C16:1)	2.05E-02	4.31E-01	−0.12 (0.05)		
*CIART*	rs322666984 (4:98.801 Mb)	Missense variant (G/C)	BFT_1_	4.07E-03	8.54E-02	−3.20 (1.12)	C	0.214
*PER1*	rs81436952 (12:53.368 Mb)	Missense variant (C/T)	LD (C18:3)	1.16E-02	1.22E-01	0.04 (0.02)	C	0.058
			GM (C18:3)	4.11E-02	2.96E-01	0.04 (0.02)		
	rs699427837 (12:53.365 Mb)	Missense variant (A/G)	LD (C18:3)	9.65E-03	1.22E-01	0.04 (0.02)	G	0.059
			GM (C18:3)	4.22E-02	2.96E-01	0.04 (0.02)		
	rs345340955 (12:53.368 Mb)	Splice region variant (A/T)	LD (C18:3)	2.33E-02	1.62E-01	0.04 (0.02)	T	0.054
			GM (C18:3)	1.33E-02	2.96E-01	0.05 (0.02)		
*PCK1*	rs320568163 (17:57.936 Mb)	Missense variant (A/G)	LD (C17:0)	2.23E-02	2.70E-01	−0.02 (0.01)	G	0.144
	rs331782052 (17:57.933 Mb)	Missense variant (A/G)	LD (C17:0)	2.57E-02	2.70E-01	−0.02 (0.01)	G	0.138

^a^SNPs in bold show associations that remained significant after correction for multiple testing; *q*-value: *q*-value calculated with the false-discovery rate (FDR) method; δ: estimated allele substitution effect and standard error (SE); A_1_: minor allele, MAF: Minor allele frequency; LD: *longissimus dorsi* muscle, GM: *gluteus medius* muscle; trait acronyms are defined in Supplementary Table 1.

The other association that remained significant after correction for multiple testing was that between rs320439526 genotype and stearic acid content (C18:0) of the LD muscle (Table 2). This polymorphism is located in the 5′ end of the *CRY2* gene, and it was annotated as having a putative stop gain effect in the former *Sus scrofa* assembly record (Sscrofa10.2). This led us to select it due to the high impact effect that the inactivation of this gene could have on the regulation of circadian clock rhythms and many other relevant metabolic processes. However, when interrogated in the last available assembly release for the porcine genome (Sscrofa11.1), this variant appeared to be located in the 5′-UTR of the *CRY2* gene. The Kruskal-Wallis ranking test for differences in median C18:0 content in the LD muscle yielded a *P*-value of 5.71E-03 (Supplementary Table 3), with homozygous TT pigs having a higher median stearic acid content (12.52%) than their CT (11.54%) and CC (11.30%) counterparts (Fig. 1C). The proportion of variance in stearic acid content in LD muscle explained by the rs320439526 genotype was 8.87% (SE = 0.04%).

### Polymorphisms in the *MIGA2* and *CRY2* genes are associated with mRNA expression levels

To gain new insights into the molecular basis of the two significant associations found (Table 2), we investigated whether the rs330779504 and the rs320439526 SNPs are associated with the mRNA expression of the *MIGA2* and *CRY2* genes, respectively. Previously reported hepatic and GM muscle microarray data sets^18,20^ were employed for this purpose (Supplementary Table 2). Analysis with the GEMMA software revealed a significant association between the rs330779504 polymorphism and *MIGA2* mRNA expression levels in the GM muscle (Table 3). Pigs inheriting the A-allele of the rs330779504 polymorphism showed a reduced *MIGA2* mRNA expression (Fig. 1B). Performance of a test based on the analysis of variance (ANOVA) confirmed the existence of statistically significant differences amongst genotypes (Supplementary Table 4). Moreover, a weak but significant association between the SNP rs330779504 and one of the probes defining liver *MIGA2* mRNA expression was also found (Table 3). With regard to the *CRY2* gene, when we performed an association analysis with the GEMMA software, the rs320439526 5′-UTR variant happened to be significantly associated with the expression of the corresponding gene in the GM muscle (Table 3). When we compared the *CRY2* mRNA levels corresponding to each one of the three rs320439526 genotypes (Fig. 1D) by using an ANOVA test, we found differences that almost reached significance (Supplementary Table 4).

**Table 3:** Associations between *MIGA2* and *CRY2* genotypes and the mRNA levels of the corresponding genes estimated with microarrays in *gluteus medius* skeletal muscle and liver samples from Duroc pigs; δ: estimated allele substitution effect and standard error (SE); A_1_: minor allele, MAF: Minor allele frequency; GM: *gluteus medius* skeletal muscle.

Gene	SNP	Type	Probe	Tissue	*P*-value	δ (SE)	A_1_	MAF
*MIGA2*	rs330779504 (1:269.360 Mb)	Splice region variant (G/A)	Ssc.19153.2.A1_at	GM	8.11E-06	−0.39 (0.08)	A	0.236
				LIVER	5.99E-01	0.05 (0.09)		
			Ssc.19153.1.S1_at	GM	2.78E-07	−0.53 (0.09)		
				LIVER	2.60E-02	0.16 (0.07)		
*CRY2*	rs320439526 (2:16.620 Mb)	5′-UTR variant (C/T)	Ssc.26267.1.S1_at	GM	3.01E-02	−0.19 (0.09)	T	0.353

### Inclusion of significant SNPs in a chromosome-wide association analysis

**Figure 2:**
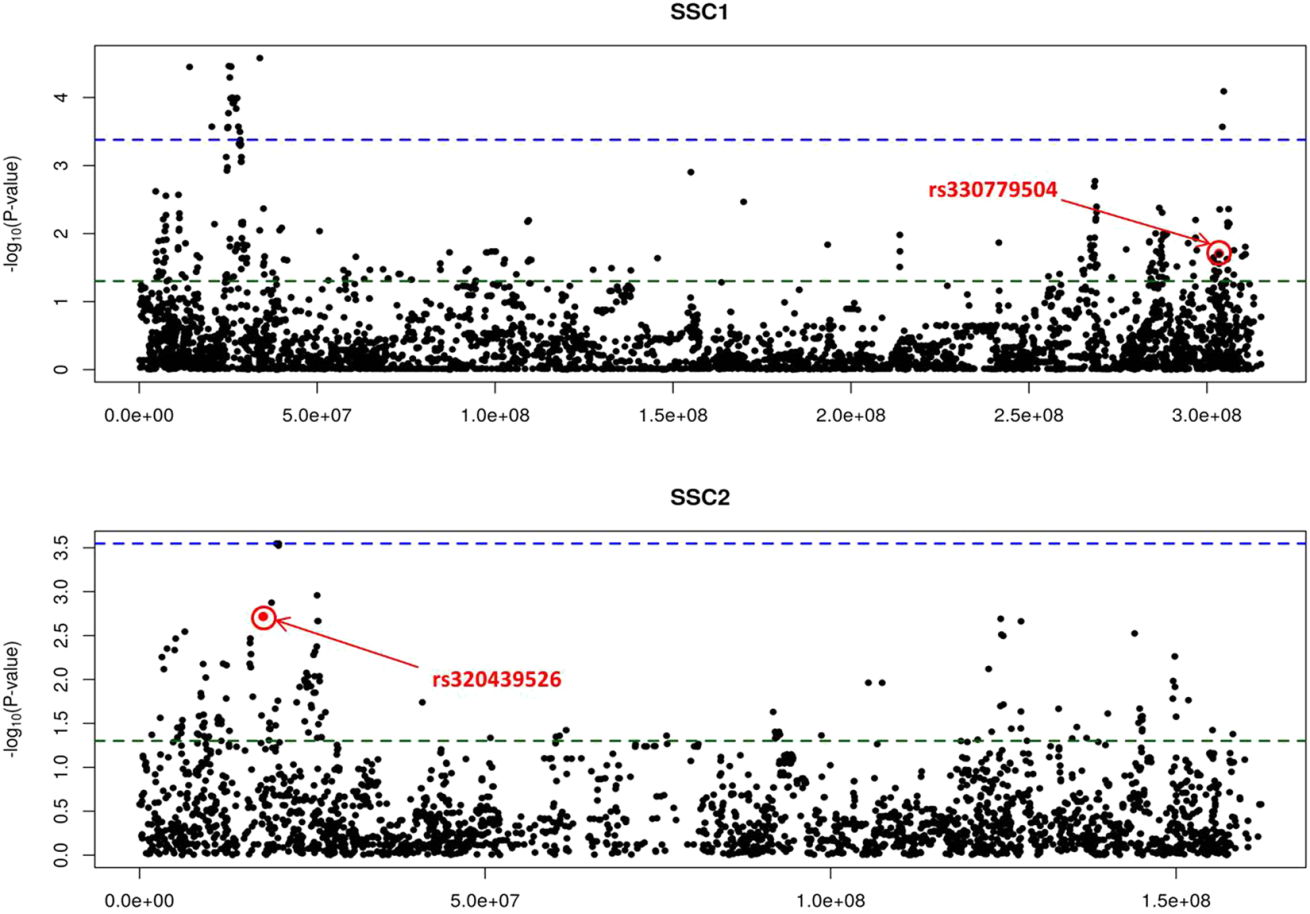
(**A**) Manhattan plot depicting the association of SNP rs330779504 and 7,188 additional SNPs mapping to pig chromosome 1 (SSC1) with serum low density lipoprotein concentration at ~190 days of age recorded in 345 Duroc pigs (Lipgen population). (**B**) Manhattan plot depicting the association of SNP rs320439526 and 3,684 additional SNPs mapping to pig chromosome 2 (SSC2) with stearic acid content in the *longissimus dorsi* muscle. The green line represents the nominal *P-value* of significance, while the blue line indicates the *P-value* of significance after correcting for multiple testing with an FDR test.

After demonstrating that in the Lipgen population the rs330779504 (*MIGA2*) and rs320439526 (*CRY2*) SNPs are associated with serum LDL concentration at 190 days and LD C18:0, respectively, we aimed to investigate whether other SNP markers located in the vicinity of rs330779504 and rs320439526 display associations with these two traits with a higher level of significance than those observed for rs330779504 and rs320439526. To achieve this goal, we merged the rs330779504 SNP with 7,188 SNPs mapping to pig chromosome 1 (SSC1) and the rs320439526 SNP with 3,684 SNPs mapping to SSC2. The SSC1 and SSC2 SNP data were extracted from Porcine SNP60 BeadChip genotyping data reported by Manunza *et al*.^18^ and González-Prendes *et al*.^21^ in the Lipgen population (Supplementary Table 2). The associations between the markers rs330779504 (*MIGA2*) and rs320439526 (*CRY2*) with LDL serum concentration at ~190 days of age and with stearic acid content in LD, respectively, were only detected at the nominal level (Fig. 2). Indeed, we did not find any significant association at the chromosome-wide level when correcting for multiple testing with the false discovery rate (FDR) approach^22^ (Fig. 2).

## Discussion

One of the main goals of our study was to evaluate whether the polymorphisms of six genes with critical roles in the regulation of the circadian rhythms (*CRY2*, *NPAS2*, *CIART*, *ARNTL2*, *PER1*, *PER2*) are associated with the variation of lipid traits recorded in 345 Duroc pigs (Lipgen population). Indeed, circadian clock genes have been broadly reported as major contributors to the regulation of lipid metabolism and maintenance of energy homeostasis^3,4,7^, driving changes in the expression of multiple transcripts and thus causing differences in protein and enzymatic activity across the day-night cycle^23,24^. We have found multiple associations between the variability of the pig circadian genes and fatty acid composition traits, but the majority of them were only significant at the nominal level (Table 2). There are reports that indicate that there is a close relationship between the activity of circadian genes and fatty acid synthesis. For instance, fatty acid elongation is under circadian control because the cyclic acetylation of acetyl-CoA synthetase 1 by the SIRT1 deacetylase modulates the intracellular concentration of acetyl-CoA^25^. Moreover, alteration of circadian genes can potentially influence liver lipid metabolism in mice^26^. With regard to the circadian genes, the only association that remained significant after correction for multiple testing was that between the rs320439526 SNP of the *CRY2* gene and C18:0 content in the *longissimus dorsi* muscle (Table 2, Supplementary Table 3). This association is particularly interesting because it has been demonstrated that the *CRY1/2* genes can repress the peroxisome proliferator-activated receptor delta (*PPARδ*) transcription factor, which has a fundamental role in lipid metabolism^15^. Indeed, the inhibition of *PPARδ* by *CRY1/2* is expected to decrease the rates of fatty acids transport and oxidation in the skeletal muscle^15^. Besides, the *CRY2* gene has multiple effects on lipid metabolism. In response to a high-fat diet, CRY-deficient mice showed an increased body weight gain despite less feed consumption compared with wild-type animals, as a result of the activation of lipogenic pathways combined with increased insulin secretion and lipid storage^27^, thus leading to obesity propensity when CRY regulatory function was disrupted. We have also shown that the rs320439526 SNP of the *CRY2* gene is associated with the expression of the *CRY2* mRNA in the *gluteus medius* muscle (Table 3, Supplementary Table 4), suggesting that this polymorphism, or a nearby mutation, has regulatory effects on the transcriptional rate of the *CRY2* gene. This polymorphism maps to the 5′-UTR of the *CRY2* gene, a region that can have broad post-transcriptional effects on gene expression by interacting with RNA-binding proteins^28^. However, the Ensembl annotation of the rs320439526 SNP does not predict any functional effect, so we favour the hypothesis that this SNP is linked to another mutation with regulatory effects on *CRY2* mRNA levels. The chromosome-wide (SSC2) association analysis depicted in Fig. 2 clearly shows that rs320439526 is not the marker displaying the most significant association with LD C18:0, thus indicating that the associations detected in our study have been produced by the existence of linkage disequilibrium between the rs320439526 SNP and a causal mutation yet to be found.

Another association that remained significant after correction for multiple testing was that between the *MIGA2* rs330779504 SNP and serum LDL concentrations at ~190 days (Table 2, Supplementary Table 3). Moreover, this SNP was also associated with *MIGA2* mRNA expression in the GM muscle and liver tissues (Table 3, Supplementary Table 4). The *MIGA2* gene, also known as *FAM73B*, and its homolog *MIGA1* (*FAM73A*) encode proteins localized to the outer membrane of mitochondria as membrane-integrated proteins and they have been previously associated with reduced body weight in mice^29^ and variations in backfat thickness in pigs^30^. In a study performed by Zhang *et al*.^10^, it was reported that MIGA1/2 proteins stabilize the dimeric complex formed by active MitoPLD, thus facilitating mitochondrial fusion^31^. Interestingly, the dynamics of mitochondrial fusion and fission is tightly related with the energy demand of cells. Indeed, nutrient abundance and starvation are associated with an increased frequency of fission and fusion events, respectively^27,32^. Besides, the capacity to produce ATP in response to changes in energy demand and supply is modulated by mitochondrial morphology^33^. A recent study reported that mitochondrial fusion induced by leptin could have important effects on the hepatic lipid accumulation^34^, but to the best of our knowledge it is currently unknown whether mitochondrial fusion/fission has any effect on cholesterol and lipoprotein metabolism. Noteworthy, the chromosome-wide analysis pictured in Fig. 2 evidenced that the association observed between the *MIGA2* rs330779504 marker and serum LDL levels at ~190 days is probably not causal, as there are some other neighboring SNPs that show more significant associations with this trait.

## Conclusions

In this work, we wanted to test whether the variability of six circadian genes (*ARNTL2*, *CIART*, *CRY2*, *NPAS2*, *PER1* and *PER2*) and two additional genes (*MIGA2* and *PCK1*) with key roles in energy homeostasis is associated with a set of lipid phenotypes recorded in Duroc pigs (Lipgen population). We have observed multiple associations between the variation of circadian genes and muscle fatty acid composition, but only that between the rs320439526 SNP of the *CRY2* gene and LD C18:0 content remained significant after correction for multiple testing. We have also detected a significant association between the rs330779504 SNP of the *MIGA2* gene and LDL concentration at 190 days. In the light of the results of the chromosome-wide analyses, we conclude that none of these two associations are causal.

## Methods

### Ethics approval

Animal care and management procedures were performed following Spanish national guidelines for the Good Experimental Practices and they were approved by the Ethical Committee of the Institut de Recerca i Tecnologia Agroalimentàries (IRTA).

### Animal material and phenotype recording

As previously reported by Gallardo *et al*.^35,36^, a total of 345 Duroc barrows belonging to 5 half-sib families and distributed in 4 fattening batches were selected from a commercial pig line, devoted to high quality meat production. This line is characterized by its high content of intramuscular fat, a feature that results in the improvement of meat juiciness and taste, hence conferring a better consumer acceptance^37^. Pigs were bred under intensive conditions of feeding and handling, and slaughtered when they reached 122 kg of live weight (~190 days of age). Phenotypic measures for different traits (Supplementary Table 1) were recorded during the productive cycle or after slaughtering: Triglycerides (TG), total cholesterol (TotalCholest), high-density lipoprotein (HDL) and low-density lipoprotein (LDL) serum concentrations at ~45 and ~190 days of age as reported by Gallardo *et al*.^17^, whereas intramuscular fat content in the LD and GM muscles and fatty acid composition for LD and GM were determined as described by Quintanilla *et al*.^19^.

### Selection and genotyping of twenty SNPs mapping to eight candidate genes

Based on the results reported by Cardoso *et al*.^1^, we took into consideration eight genes that showed differential expression before and after feeding and that, moreover, play important roles in metabolism and circadian clock regulation (Table 1). The variability of these 8 genes was characterized by using, as a source of information, RNA-seq data results from 52 Duroc pigs retrieved from the same population analyzed herewith (Supplementary Table 2). Single nucleotide polymorphisms within selected genes were retrieved among variant calling results from sequences generated by Cardoso *et al*.^16^. Variant discovery analyses were performed by following the *GATK Best Practices workflow for SNP calling* (https://software.broadinstitute.org/gatk/documentation/article.php?id=3891) on RNA-seq data. Briefly, after read mapping, sequences were split into exon segments and intronic overhanging sequences hard-clipped. Mapping qualities were reassigned by using the SplitNCigarReads GATK tool (https://software.broadinstitute.org/gatk), and the Haplotype Caller tool (https://software.broadinstitute.org/gatk) was used to detect SNPs for each analyzed sample (N = 52). Variant effect prediction on detected polymorphisms was estimated by using the SnpEff software^38^ and those that showed potential functional or regulatory effects (*i*.*e*. high impact, missense, splice site regions, 5′-UTR) within selected genes were kept for genotyping. Moreover, we also selected 3 SNPs showing potential functional or regulatory effects (rs322533788, rs335603631 and rs326158774) that were retrieved from the Ensembl database (https://www.ensembl.org). A total of 20 selected SNPs and their flanking sequences (60 nucleotides upstream and downstream), were submitted to the Custom TaqMan Assay Design Tool website (https://www5.appliedbiosystems.com /tools/cadt/; Life Technologies) to ascertain if they were amenable to genotyping in a TaqMan Open Array multiplex assay platform. Genotyping was performed at the Servei Veterinari de Genètica Molecular at the Universitat Autònoma de Barcelona (http://sct.uab.cat/svgm/en) by using a QuantStudio 12 K flex Real-Time PCR System (ThermoFisher Scientific).

### Association analyses between twenty selected SNPs and porcine lipid-related traits

The PLINK software^39^ was used for processing genotyped data. Association analyses between genotyped polymorphisms and phenotypes were performed with the Genome wide efficient mixed-model association (GEMMA) software^40^. This package uses a mixed model approach to account for population stratification and relatedness by calculating a genomic kinship matrix with SNPs genotypes as random effects and provides an exact test of significance. We implemented a univariate mixed model as follows:

$$y=W\alpha +x\delta +u+\varepsilon $$

where ***y*** is the vector of phenotypic observations for every individual; ***α*** corresponds to a vector including the intercept plus the fixed effects, *i*.*e*. batch effect with 4 categories (all traits), farm origin effect with 3 categories (all traits), data of extraction with 2 categories within batch (only for TotalCholest, TG, HDL and LDL serum concentration, that were measured at approximately 45 and 190 days). The ***α*** vector also contains the regression coefficients of the following covariates: live weight at slaughterhouse for TotalCholest, TG, HDL and LDL serum concentrations, and IMF content in LD and GM for LD and GM fatty acid composition respectively; ***W*** is the incidence matrix relating phenotypes with the corresponding effects; ***x*** is the vector of the genotypes corresponding to the set of selected polymorphisms; ***δ*** is the allele substitution effect for each polymorphism; ***u*** is a vector of random individual effects with a n-dimensional multivariate normal distribution MVN_n_ (0, λ *τ*^−1^ K), where *τ*^−1^ is the variance of the residual errors, λ is the ratio between the two variance components and K is a known relatedness matrix derived from the SNPs; and ***ε*** is the vector of residual errors.

The association between lipid-related traits and the twenty analysed polymorphisms was assessed on the basis of the estimated allele substitution effects. The significance of these effects was established by implementing a correction for multiple testing using the FDR method reported by Benjamini and Hochberg^22^. Moreover, we compared the phenotypic medians corresponding to each one of the three possible genotypes by applying the non-parametric Kruskal-Wallis test, due to the non-normal data distribution of lipid phenotypes under study.

### Association analyses between the *rs330779504* and *rs320439526* polymorphisms and the expression of the genes that contain them

*Gluteus medius* skeletal muscle and liver samples were collected from 103 Duroc pigs belonging to the Lipgen population. Samples were retrieved after slaughtering, and immediately frozen at −80 °C in liquid nitrogen. Total RNA was isolated from GM samples by using the TRIzol method^41^ and the RiboPure kit (Ambion, Austin, TX) following manufacturer’s recommendations. Transcriptomic mRNA expression profiles were then assessed by hybridization to the GeneChip Porcine arrays (Affymetrix Inc., Santa Clara, CA), as previously reported by Cánovas *et al*.^20^. Expression data corresponding to GM muscle and liver samples are deposited in NCBI’s Gene Expression Omnibus^42^ and can be accessed through GEO Series accession number GSE115484 (https://www.ncbi.nlm.nih.gov/geo/query/acc.cgi?acc=GSE115484). Data pre-processing, background correction, normalization and log-transformation of expression values between samples were carried out by computing a Robust Multi-array Average (RMA) as described by Irizarry *et al*.^43^.

The correspondence between genes and microarray expressed probes was assessed with the Biomart database available at Ensembl repositories (https://www.ensembl.org/biomart/martview/). Expression levels for selected genes were then extracted from microarray samples for both GM muscle and liver tissues and used as continuous variables in association analyses, following the same statistical model previously described for phenotype records and correcting for batch (4 categories), farm of origin (3 categories) and laboratory (2 categories) as fixed effects. Moreover, we compared the phenotypic means corresponding to each one of the three possible genotypes by applying an ANOVA test.

### Inclusion of the *MIGA2* rs330779504 and *CRY2* rs320439526 SNPs in a chromosome-wide association analysis

As previously described by Manunza *et al*.^18^ and González-Prendes *et al*.^21^, the population employed in the current experiment was typed with the Porcine SNP60 BeadChip (Illumina, San Diego, CA) which contains probes for 62,163 SNPs (Supplementary Table 2). The GenomeStudio software (Illumina) was used for quality control analyses, as reported by Manunza *et al*.^18^. The PLINK software^39^ was used for removing SNPs that (a) did not map to autosomal chromosomes, (b) had minor allele frequency (MAF) <0.05, (c) with rate of missing genotypes >0.05, (d) major departures from the Hardy-Weinberg equilibrium (*P*-value = 0.001), (e) had a GenCall score <0.15, (f) had a call rate <0.95, or (g) that could not be mapped to the pig reference genome. A total of 36,710 SNPs were finally retrieved after filtering and merged with genotyping data corresponding to the rs330779504 and the rs320439526 SNPs. Association analyses were performed with the GEMMA software^40^ as described before, and multiple testing correction was implemented with the FDR method^22^ by establishing a chromosome-wide threshold of significance.

## Supplementary information

Supplementary Materials

## Data Availability

Expression data corresponding to GM muscle and liver samples are deposited at NCBI’s Gene Expression Omnibus and are accessible through GEO Series accession number GSE115484 (https://www.ncbi.nlm.nih.gov/geo/query/acc.cgi?acc=GSE115484). Genotypes and phenotypes for the 345 Duroc pigs (Lipgen population) have been deposited in the Figshare public repository (https://figshare.com/s/2e636697009360986794).
