# Supplementary material for: Polymorphisms of the cryptochrome 2 and mitoguardin 2 genes are associated with the variation of lipid-related traits in Duroc pigs
Source: Sci Rep. 2019 Jun 21;9:9025. doi: 10.1038/s41598-019-45108-z (PMC6588565; doi:10.1038/s41598-019-45108-z)
Supplement: Supplementary file 1 — Supplementary Materials [file 41598_2019_45108_MOESM1_ESM.docx]

**Polymorphisms of the cryptochrome 2 and mitoguardin 2 genes are associated with the variation of lipid-related traits in Duroc pigs**

^1^Emilio Mármol-Sánchez, ^2^Raquel Quintanilla, ^1,3^Taina F. Cardoso, ^4^Jordi Jordana and ^1,4^*Marcel Amills

^1^Department of Animal Genetics, Centre for Research in Agricultural Genomics (CRAG), CSIC-IRTA-UAB-UB, Campus de la Universitat Autònoma de Barcelona, Bellaterra, Spain.

^2^Animal Breeding and Genetics Programme, Institute for Research and Technology in Food and Agriculture (IRTA), Torre Marimon, Caldes de Montbui, Spain.

^3^CAPES Foundation, Ministry of Education of Brazil, Brasilia, D. F., Brazil.

^4^Departament de Ciència Animal i dels Aliments, Universitat Autònoma de Barcelona, Bellaterra, Spain.

*[marcel.amills@uab.cat](mailto:marcel.amills@uab.cat)

**Supplementary Table 1:** Phenotypes measured in 345 Duroc pigs belonging to the Lipgen population. LD: *longissimus dorsi* skeletal muscle; GM: *gluteus medius* skeletal muscle.

| **Trait** | **Units** | **Mean** | **SD** | **Name** |
| --- | --- | --- | --- | --- |
| **TotalCholest_1_** | mg/dL | 80.4763 | 25.5029 | Total cholesterol serum concentration at ~45 days of age |
| **HDL_1_** | mg/dL | 31.4790 | 9.6696 | High-density lipoprotein serum concentration at ~45 days of age |
| **LDL_1_** | mg/dL | 40.4393 | 16.4027 | Low-density lipoprotein serum concentration at ~45 days of age |
| **TG_1_** | mg/dL | 42.6805 | 18.4389 | Triglycerides serum concentration at ~45 days of age |
| **TotalCholest_2_** | mg/dL | 125.0541 | 26.1000 | Total cholesterol serum concentration at ~190 days of age |
| **HDL_2_** | mg/dL | 51.8844 | 9.9040 | High-density lipoprotein serum concentration at ~190 days of age |
| **LDL_2_** | mg/dL | 62.7.373 | 20.7480 | Low-density lipoprotein serum concentration at ~190 days of age |
| **TG_2_** | mg/dL | 51.7.420 | 23.3554 | Triglycerides serum concentration at ~190 days of age |
| **BFT_3-4_** | mm | 33.6207 | 6.3174 | Back fat thickness between the 3^rd^ and 4^th^ rib |
| **BFT_last_** | mm | 38.4075 | 11.2738 | Back fat thickness at the last rib |
| **BFT_1_** | mm | 43.5646 | 8.0844 | Back fat thickness at the first rib |
| **LDIMF** | % | 3.8973 | 1.4883 | Intramuscular fat in LD |
| **GMIMF** | % | 51.909 | 2.0358 | Intramuscular fat in GM |
| **LDCholest** | mg/g | 58.5760 | 9.2335 | Total cholesterol in LD |
| **GMCholest** | mg/g | 64..583 | 11.0147 | Total cholesterol in GM |
| **LD(C10:0)** | % | 0.0949 | 0.0666 | Capric acid in LD |
| **LD(C12:0)** | % | 0.0844 | 0.0393 | Lauric acid in LD |
| **LD(C14:0)** | % | 1.3546 | 0.2768 | Myristic acid in LD |
| **LD(C16:0)** | % | 23.4100 | 1.6388 | Palmitic acid in LD |
| **LD(C16:1)** | % | 2.9587 | 0.5883 | Palmitoleic acid in LD |
| **LD(C17:0)** | % | 0.2074 | 0.081 | Margaric acid in LD |
| **LD(C18:0)** | % | 11.7254 | 1.2208 | Stearic acid in LD |
| **LD(C18:1)** | % | 34.8465 | 5.2031 | Oleic acid in LD |
| **LD(C18:1 n-7)** | % | 4.2845 | 0.3452 | Vaccenic acid in LD |
| **LD(C18:2)** | % | 14.2666 | 5.1459 | Linoleic acid in LD |
| **LD(C18:3)** | % | 0.1508 | 0.095 | α-Linolenic acid in LD |
| **LD(C20:0)** | % | 0.1751 | 0.0765 | Arachidic acid in LD |
| **LDSFA** | % | 37.0523 | 2.4196 | Saturated fatty acids in LD |
| **LDUFA** | % | 62.9536 | 2.4173 | Unsaturated fatty acids in LD |
| **LDPUFA** | % | 19.7941 | 7.4273 | Polyunsaturated fatty acids in LD |
| **LDMUFA** | % | 43.1594 | 5.7051 | Monounsaturated fatty acids in LD |
| **LDPUFA/MUFA** | ratio | 0.4935 | 0.2734 | Polyunsaturated/Monounsaturated fatty acid ratio in LD |
| **LDFAn6** | % | 18.8829 | 7.1991 | Omega-6 fatty acids in LD |
| **LDFAn3** | % | 0.9117 | 0.2848 | Omega-3 fatty acids in LD |
| **LDFAn6/FAn3** | ratio | 20.6623 | 4.7865 | Omega-6/Omega-3 fatty acids ratio in LD |
| **GM(C10:0)** | % | 0.1056 | 0.0629 | Capric acid in GM |
| **GM(C12:0)** | % | 0.0901 | 0.0361 | Lauric acid in GM |
| **GM(C14:0)** | % | 1.3839 | 0.2289 | Myristic acid in GM |
| **GM(C16:0)** | % | 23.2136 | 1.4139 | Palmitic acid in GM |
| **GM(C16:1)** | % | 0.286 | 0.0515 | Palmitoleic acid in GM |
| **GM(C17:0)** | % | 0.2775 | 0.1662 | Margaric acid in GM |
| **GM(C18:0)** | % | 11.1852 | 1.1228 | Stearic acid in GM |
| **GM(C18:1)** | % | 35.0596 | 4.5208 | Oleic acid in GM |
| **GM(C18:1 n-7)** | % | 4.0568 | 0.3103 | Vaccenic acid in GM |
| **GM(C18:2)** | % | 15.0198 | 4.1459 | Linoleic acid in GM |
| **GM(C18:3)** | % | 0.1582 | 0.0935 | α-Linolenic acid in GM |
| **GM(C20:0)** | % | 0.1749 | 0.1183 | Arachidic acid in GM |
| **GMSFA** | % | 36.4309 | 2.0786 | Saturated fatty acids in GM |
| **GMUFA** | % | 63.5689 | 2.0786 | Unsaturated fatty acids in GM |
| **GMPUFA** | % | 20.4620 | 6.1247 | Polyunsaturated fatty acids in GM |
| **GMMUFA** | % | 43.1071 | 4.9629 | Monounsaturated fatty acids in GM |
| **GMPUFA/MUFA** | ratio | 0.4992 | 0.2191 | Polyunsaturated/Monounsaturated fatty acid ratio in GM |
| **GMFAn6** | % | 19.3557 | 5.8878 | Omega-6 fatty acids in GM |
| **GMFAn3** | % | 1.1066 | 0.3535 | Omega-3 fatty acids in GM |
| **GMFAn6/FAn3** | ratio | 17.7628 | 3.7950 | Omega-6/Omega-3 fatty acids ratio in GM |

**Supplementary Table 2:** Sources of the experimental data described in the current work.

| **Type of data** | **Source** |
| --- | --- |
| Genotyping of 20 SNPs corresponding to 8 genes in 345 Duroc pigs | Current work |
| Lipid serum phenotypes (cholesterol, LDL, HDL, triglycerides) measured in 345 Duroc pigs at 2 time points (45 days and 190 days of age) | Gallardo, D. *et al.* Mapping of quantitative trait loci for cholesterol, LDL, HDL, and triglyceride serum concentrations in pigs. *Physiol. Genomics* 35, 199–209 (2008).  Manunza, A. *et al.* A genome-wide association analysis for porcine serum lipid traits reveals the existence of age-specific genetic determinants. *BMC Genomics* 15, 758 (2014). |
| Intramuscular fat content and composition phenotypes measured in 345 Duroc pigs in two skeletal muscles (*longissimus dorsi* and *gluteus medius*) | Quintanilla, R. *et al.* Porcine intramuscular fat content and composition are regulated by quantitative trait loci with muscle-specific effects. *J. Anim. Sci.* 89, 2963–71 (2011). |
| RNA-Seq data from *gluteus medius* muscle measured in 52 Duroc pigs | Cardoso, T. F. *et al.* RNA-seq based detection of differentially expressed genes in the skeletal muscle of Duroc pigs with distinct lipid profiles. *Sci. Rep.* 7, 40005 (2017). |
| Microarray data from *gluteus medius* measured in 103 Duroc pigs | Cánovas, A. *et al.* Segregation of regulatory polymorphisms with effects on the gluteus medius transcriptome in a purebred pig population. *PLoS One* **7,** e35583 (2012). |
| Microarray data from liver measured in 103 Duroc pigs | Manunza, A. *et al.* A genome-wide association analysis for porcine serum lipid traits reveals the existence of age-specific genetic determinants. *BMC Genomics* 15, 758 (2014). |
| Porcine SNP60 BeadChip data from 3345 Duroc pigs | Manunza, A. *et al.* A genome-wide association analysis for porcine serum lipid traits reveals the existence of age-specific genetic determinants. *BMC Genomics* 15, 758 (2014).  González-Prendes, R. *et al.* Joint QTL mapping and gene expression analysis identify positional candidate genes influencing pork quality traits. *Sci. Rep.* **7,** 39830 (2017). |

**Supplementary Table 3:** Genotypic medians corresponding to two *MIGA2* and *CRY2* polymorphisms. Median ± SE: Median values ± standard error for LDL Cholesterol serum concentration (mg/dL) and stearic acid content (%) in the *longissimus dorsi* skeletal muscle. KW *P*-value: Nominal *P-*value obtained with the Kruskal-Wallis test.

| **Gene** | **SNP** | **Type** | **Trait** | **Genotype** | **Median ± SE** | **KW *P*-value** |
| --- | --- | --- | --- | --- | --- | --- |
| *MIGA2* | rs330779504 (1:269.360 Mb) | Splice region variant (G/A) | LDL_2_ | GG  (N=191) | 58.4 ± 1.25 | 5.14E-03 |
|  |  |  |  |  |  |  |
|  |  |  |  | GA  (N=125) | 61.75 ± 2.19 |  |
|  |  |  |  |  |  |  |
|  |  |  |  | AA  (N=16) | 69.35 ± 5.08 |  |
|  |  |  |  |  |  |  |
| *CRY2* | rs320439526 (2:16.620 Mb) | 5'-UTR variant (C/T) | LD(C18:0) | CC  (N=135) | 11.3 ± 0.09 | 5.71E-03 |
|  |  |  |  |  |  |  |
|  |  |  |  | CT  (N=161) | 11.54 ± 0.10 |  |
|  |  |  |  |  |  |  |
|  |  |  |  | TT  (N=37) | 12.52 ± 0.24 |  |
|  |  |  |  |  |  |  |

**Supplementary Table 4:** Normalized probe expression values for *MIGA2* and *CRY2* genotypes. Mean ± SE: Mean values ± standard error for estimated normalized probe expression values; ANOVA *P*-value: Nominal *P*-value obtained with an ANOVA test.

| **Gene** | **SNP** | **Type** | **Probe** | **Genotype** | **Mean ± SE** | **ANOVA *P*-value** |
| --- | --- | --- | --- | --- | --- | --- |
| *MIGA2* | rs330779504 (1:269.360 Mb) | Splice region variant (G/A) | Ssc.19153.1.S1_at | GG (N=48) | 8.64 ± 0.07 | 4.81E-09 |
|  |  |  |  |  |  |  |
|  |  |  |  | GA (N=33) | 7.98 ± 0.08 |  |
|  |  |  |  |  |  |  |
|  |  |  |  | AA  (N=6) | 7.78 ± 0.18 |  |
|  |  |  |  |  |  |  |
| *CRY2* | rs320439526 (2:16.620 Mb) | 5'-UTR variant (C/T) | Ssc.26267.1.S1_at | CC  (N=37) | 8.46 ± 0.08 | 6.30E-02 |
|  |  |  |  |  |  |  |
|  |  |  |  | CT  (N=45) | 8.22 ± 0.06 |  |
|  |  |  |  |  |  |  |
|  |  |  |  | TT  (N=6) | 8.21 ± 0.12 |  |
|  |  |  |  |  |  |  |
